# *Slc12a2* loss in insulin-secreting β-cells links development of overweight and metabolic dysregulation to impaired satiation control of feeding

**DOI:** 10.1152/ajpendo.00197.2023

**Published:** 2023-10-11

**Authors:** Yakshkumar Dilipbhai Rathod, Rana Abdelgawad, Christian A. Hübner, Mauricio Di Fulvio

**Affiliations:** ^1^Department of Pharmacology and Toxicology, School of Medicine, Wright State University, Dayton, Ohio, United States; ^2^Institut für Humangenetik Am Klinikum 1, Universitätsklinikum Jena, Jena, Germany

**Keywords:** β-cell, metabolic syndrome, overweight/obesity, satiation, satiety, Slc12a2

## Abstract

Male mice lacking the Na^+^-K^+^-2Cl^–^ cotransporter *Slc12a2* (*Nkcc1*) specifically in insulin-secreting β-cells (*Slc12a2^βKO^*) have reduced β-cell mass and mild β-cell secretory dysfunction associated with overweight, glucose intolerance, insulin resistance, and metabolic abnormalities. Here, we confirmed and extended previous results to female *Slc12a2^βKO^* mice, which developed a similar metabolic syndrome-like phenotype as males, albeit milder. Notably, male and female *Slc12a2^βKO^* mice developed overweight without consuming excess calories. Analysis of the feeding microstructure revealed that young lean *Slc12a2^βKO^* male mice ate meals of higher caloric content and at a relatively lower frequency than normal mice, particularly during the night. In addition, overweight *Slc12a2^βKO^* mice consumed significantly larger meals than lean mice. Therefore, the reduced satiation control of feeding precedes the onset of overweight and is worsened in older *Slc12a2^βKO^* mice. However, the time spent between meals remained intact in lean and overweight *Slc12a2^βKO^* mice, indicating conserved satiety responses to ad libitum feeding. Nevertheless, satiety was intensified during and after refeeding only in overweight males. In lean females, satiety responses to refeeding were delayed relative to age- and body weight-matched control mice but normalized in overweight mice. Since meal size did not change during refeeding, these data suggested that the satiety control of eating after fasting is impaired in lean *Slc12a2^βKO^* mice before the onset of overweight and independently of their reduced satiation responses. Therefore, our results support the novel hypothesis that reduced satiation precedes the onset of overweight and the development of metabolic dysregulation.

**NEW & NOTEWORTHY** Obesity, defined as excess fat accumulation, increases the absolute risk for metabolic diseases. Although obesity is usually attributed to increased food intake, we demonstrate that body weight gain can be hastened without consuming excess calories. In fact, impaired meal termination control, i.e., satiation, is detectable before the development of overweight in an animal model that develops a metabolic syndrome-like phenotype.

## Graphical abstract

**Figure:**
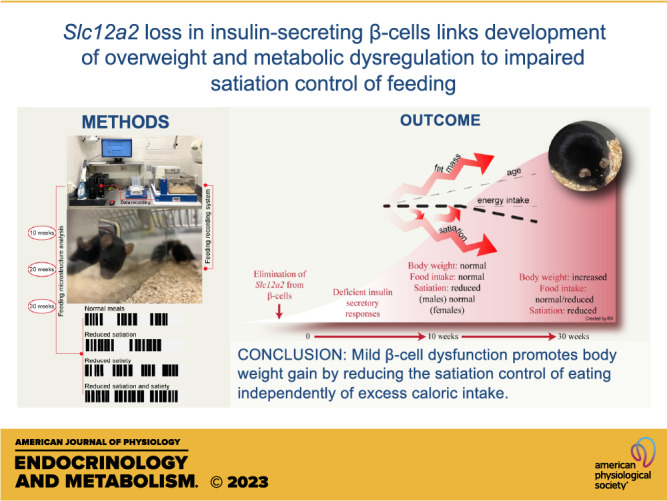

## INTRODUCTION

Disrupted insulin responses to food, overfeeding, and overweight/obesity all contribute to the onset and progression of insulin resistance and metabolic syndrome, which eventually may lead to β-cell failure and type-2 diabetes (T2D) (1). However, the role of reduced β-cell mass and/or impaired insulin responses on feeding behavior and its relationship to body weight (BW) gain and insulin resistance have been difficult to disentangle (2). Indeed, if one considers the hypothesis that hyperinsulinemia is a compensatory mechanism against insulin resistance, in turn, a consequence of overfeeding, then the latter may drive obesity-associated metabolic syndrome (3) and T2D (4). However, overfeeding can precede hyperinsulinemia and insulin resistance (5, 6) whereas hyperinsulinemia and insulin resistance may coexist before the onset of obesity (7, 8).

The pathogenesis of obesity, insulin resistance, and abnormal insulin responses to food are all interlinked (9). In fact, lowering insulin responses to high-fat diets (HFD) reduces BW gain independently of reduced food intake (10). Further, the progression of HFD-induced overweight/obesity and the metabolic syndrome are both attenuated by pharmacological or genetic reduction of plasma insulin responses to feeding or by inhibition of insulin signaling, without evident changes in net daily energy intake (11–15). These results suggest that insulin responses may play an important role in the modulation of BW gain by mechanisms not directly or exclusively related to net quantitative changes in calorie consumption. Insulin is known to directly limit food intake by engaging complex central mechanisms (16), which appear dysregulated under insulin-resistant conditions (17) or in the context of chronic hyperinsulinemia (18, 19). This is expected if one considers that reduced and/or absent central insulin signaling promotes hyperphagia and reduces energy metabolism (20, 21). Similarly, the behavioral control of energy intake appears compromised in obesity, metabolic syndrome, prediabetes states, T2D, and in patients with neurodegenerative diseases, even before changes in daily energy intake are observed (22–32).

Therefore, it remains unknown whether the onset of overweight and its associated metabolic consequences are triggered by abnormal feeding behavior, rather than positive changes in net food intake in turn consequence of reduced insulin secretory responses to food or if they are pathological results of overweight. Early experiments have suggested a role for portal insulin in the satiation control of food intake, i.e., meal size regulation (33). In addition, recent data demonstrated that prandial insulin responses are associated with reduced meal size, i.e., increase satiation, in lean 10-wk-old C57BL/6J mice without reducing net calorie intake (34). Therefore, deficient or altered insulin responses to food may impact short/long-term satiation control of meal termination. In line, reduced insulin responses to food in rats and humans treated with diazoxide resulted in decreased energy intake (35, 36). However, mice lacking K_ATP_ channels, which do not produce the first phase of insulin secretion, showed compensatory secretory responses to refeeding resulting in apparently normal food intake (37). Although feeding behavior or the dynamics of food intake were not measured in these mice, a reduction in the satiation control of food intake, i.e., increased meal size, is expected to sustain robust insulin responses to feeding. Consequently, these data together support the hypothesis that meal size can be under the control, at least partially, of islet β-cell function and that both may play a physiologically relevant role in the maintenance of BW, even in the absence of increased daily calorie intake. This is conceptually important; energy intake is invariably the product between meal size and its frequency (38), but only meal size appears strongly associated with hastened BW gain, overweight, and obesity (39, 40).

We have recently shown that mice lacking the ubiquitous Na^ +^-K^+^-2Cl^–^ cotransporter-1 (*Nkcc1*, *Slc12a2*), specifically in insulin-secreting β-cells, develop overweight, nonalcoholic steatohepatitis, and a metabolic syndrome-like phenotype associated with reduced β-cell mass, mild secretory dysfunction, and reduced calorie consumption per unit of BW (41). Therefore, given the functional role that *Slc12a2* plays in the secretory response (42), here we tested the hypothesis that impaired satiation responses to ad libitum feeding precede the onset of BW gain in these mice. Our results demonstrate that lean *Slc12a2^βKO^* mice have reduced satiation responses to feeding, which worsens in overweight mice, particularly in males, thus suggesting that impaired behavioral control of eating may play a potentially causative role in the development of overweight and metabolic syndrome.

## MATERIALS AND METHODS

### Animals

Male and female mice (10, 20, and 30 wk old) were congenic on the C57BL/6J genetic background (RRID:IMSRJAX:000664) and crossed for ∼10 generations. *Slc12a2^lox/lox^* mice were provided by Dr. Christian A. Hübner (Jena University, Jena, Germany) and mated to *Ins1^Cre^* mice [Jackson Labs stock 026801, B6(Cg)-*Ins1^tm1.1(cre)Thor^*/J] constitutively expressing *Cre* recombinase only in islet β-cells (43) to generate homozygous *Ins1^Cre^:Slc12a2^lox/lox^* mice (*Slc12a2^βKO^*). As a control, we used *Ins1^Cre^* mice of both sexes.

We have verified and extensively confirmed that *Slc12a2^βKO^* mice lack *Slc12a2* exclusively in insulin-secreting β-cells (41). Male and female mice were studied following the Animal Care Committee protocols approved by Wright State University. All methods involving mice were carried out in accordance with relevant guidelines and regulations.

### Plasma Biochemistry, Blood Glucose, and Tolerance Tests

Plasma was obtained from 6-h or 16-h fasted mice (0730–1330 h or 1600–0800 h, respectively) or at 1330–1400 h from mice fed ad libitum. Plasma triglycerides and glycerol were quantified by using commercial kits (no. 10010303 and 10010755, respectively, Cayman, Ann Arbor, MI). Plasma insulin and blood glucose were determined by ELISA (10-1247-01, Mercodia, Winston-Salem, NC) and by using a glucometer (FreeStyle-Lite, Abbott, IL), respectively. Glucose and insulin tolerance tests were performed as we have described (44).

### Tissue Processing, Morphometry Analysis, and Body Composition

Mice were deeply anesthetized (Euthasol; 150 mg/kg, ip), transcardially perfused with ice-cold PBS/heparin (0.1 mM/10 U/mL, pH 7.4) followed by 4% paraformaldehyde fixative to euthanize them, and tissues were collected essentially as described previously (45). Tissue embedding and sectioning were done at AML Laboratories (Saint Augustine, FL). Tissue sections were blindly processed for hematoxylin-eosin (H&E)-staining (no. HHS16, Sigma-Aldrich; 1% phloxine B no. 19350 Certified Generon plus eosin Y no. SE23-500D, Fisher Chemical) and mounted (Permount SP15-100, Fisher Science, Waltham MA) to capture digital images through a digital camera attached to a Nikon Eclipse 600 microscope (Nikon Corp., Japan). Adipose tissue morphometry was performed by using Fiji's (imagej.net/software/fiji, v2.14.0/1.54f) Adiposoft plug-in (imagej.net/plugins/adiposoft) on H&E-stained retroperitoneal fat tissue sections at ×200 magnification. Surface area data (pixels^2^) were manually transformed to micrometers squared after calibration against a 50-µm ruler (2.28 pixels/µm, 5.2 pixels^2^/µm^2^). The results were then confirmed by applying the automatic measuring function of the plug-in. The total body fat and water and lean mass of mice were determined by using the whole body quantitative magnetic resonance analyzer EchoMRI-500 system (EchoMRI LLC, Houston TX) as indicated (46).

### Energy Intake and Meal Definition

Mice had ad libitum access to water and standard chow (Teklad 22/5 Rodent Diet no. 8640, Envigo; 3.0 kCal/g). When mice were fasted, only water was provided. Housing conditions were set as 12:12-h light (0630–1830 h) and dark (1830–0630 h) cycles with an ambient temperature of ∼22°C. Experiments were performed using male and female mice from ∼8 to ∼35 wk of age housed in groups. Food intake and feeding microstructure were simultaneously monitored and recorded during 2 consecutive weeks in 10- to 30-wk-old mice after a week of acclimation in the metabolic cage (Feed and Water Intake Activity Monitor System HM-2, MBRose, Faaborg, Denmark). The overall settings, calibration, and design of these experiments have been described in detail elsewhere (47). By definition, a meal consists of a cluster of feeding events/bouts separated by short intervals of time or interbout intervals. This cluster of feeding events/bouts, which constitute a single meal, is separated from the next cluster of feeding events by a minimum intermeal interval (IMI) threshold of 5 min (47). We adopted a minimum meal size of 0.050 g as the lowest amount of food taken in bouts clustered within 5 min. Since no maximal meal size was imposed, a single meal is therefore recorded when the total weight of all single feeding events/bouts is ≥0.050 g and clustered within 5 min of the next cluster of feeding events. Therefore, the feeding microstructure of mice was analyzed based on the above definition of a meal and the behavioral definitions of satiation (meal termination) determined by the size (kCal) of a meal, and satiety (time spent between meals) determined by the minimum IMI (5 min). In addition to the mean caloric content of meals and IMI, the following parameters were recorded and contextually analyzed: meal frequency (the number of meals in a defined period of time: nocturnal, diurnal, after fasting, etc.), meal duration (the mean time mice spend eating a single meal), and feeding rate (meal size/meal duration).

### Fasting Protocol

Mice BW was recorded before fasting at 1600 h and immediately before allowing refeeding at 0800 h to record BW loss during 16 h of fasting (equivalent to ∼75% reduction in net daily energy intake). Net BW regain, energy intake, and eating behavior (feeding microstructure) were continuously monitored for 24 h during refeeding to evaluate the kinetics of fasting-induced hyperphagia and satiety responses, as described previously (47).

### Statistics

Results are represented as the means ± SE, with the number of individual points (*n*) indicated. Statistical significance for a *P* value <0.05 between groups was obtained by applying one-way or two-way analyses ANOVA, as appropriate, followed by the Tukey-Kramer post hoc test. Grubbs’ tests were applied to detect single outliers. Statistical analyses were conducted by using GraphPad Prism v7 (RRID:SCR002798, GraphPad Software Inc., San Diego, CA). Normal distribution and homogeneity of data variance were tested using Shapiro-Wilk and *F* tests, respectively. A priori exclusion criteria were data from mice that died before 35 wk of age preventing data collection at any time point during experiments.

## RESULTS

### Overweight, Glucose Intolerance, Insulin Resistance, and Dyslipidemia in Female *Slc12a2^βKO^* Mice

**Figure 1.**
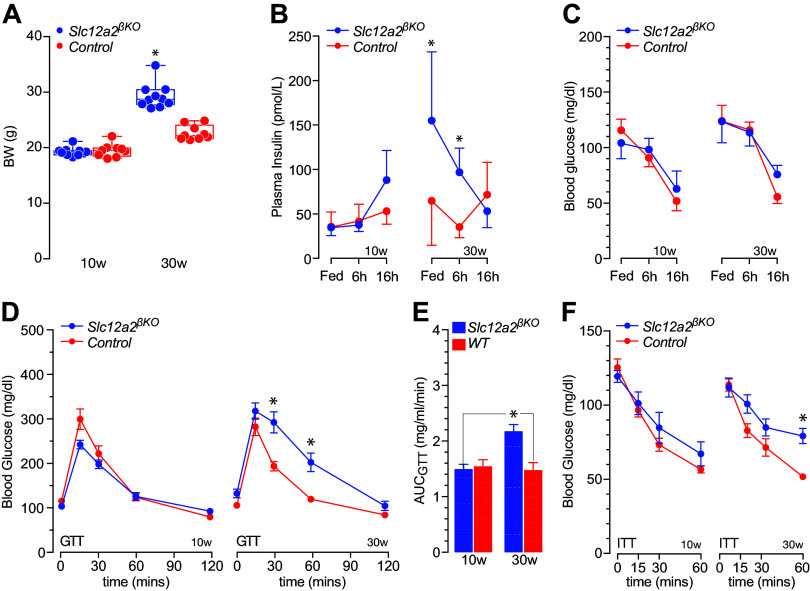
Absolute body weight, plasma insulin, blood glucose, glucose tolerance, and insulin sensitivity of *Slc12a2^βKO^* mice. *A*: body weight (BW) of 10-wk-old (10w) and 30-wk-old (30w) female *Slc12a2^βKO^* and control mice fed ad libitum a chow diet. Each dot represents the absolute BW of a single mouse, distributed as upper and lower quartiles (box) with the lowest and highest values (whiskers). The means (horizontal bars) and their statistical significance are noted (*n* = 9–10; *P < 0.01). *B* and *C*: plasma insulin (*B*; pmol/L) and blood glucose (*C*; mg/dL) of 10- and 30-wk-old female *Slc12a2^βKO^* and control mice either fed or after 6 h or 16 h of fasting (means ± SE; *n* > 3; *P < 0.01). *D* and *E*: blood glucose (mg/dL) excursions during glucose tolerance tests (GTT; *D*) performed in 10- and 30-wk-old female *Slc12a2^βKO^* and control mice after 6 h of fasting (means ± SE; *n* = 8–11; *P < 0.05) and the areas under the curve (AUC; *E*; mg/mL/min) of GTT responses (means ± SE). *F*: blood glucose responses to exogenous insulin performed in 6-h fasted *Slc12a2^βKO^* and control mice of 10 wk and 30 wk of age. ITT, insulin tolerance test. Each point represents the means ± SE (*n* = 9–11; *P < 0.05).

**Figure 2.**
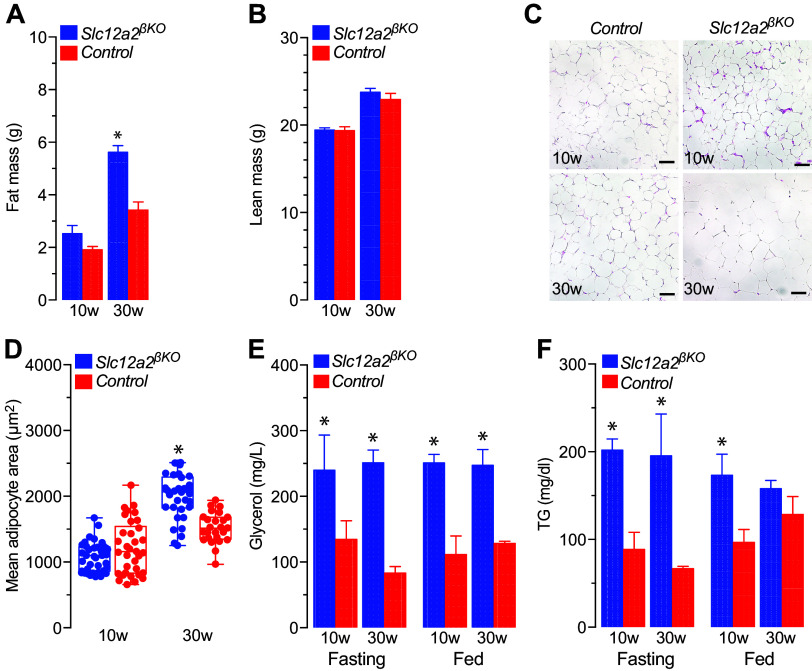
Body composition fat tissue histology and morphometry, plasma glycerol, and triglycerides (TG) of *Slc12a2^βKO^* mice. *A* and *B*: net fat (*A*; g) and lean (*B*; g) mass of 10-wk-old (10w) and 30-wk-old (30w) female *Slc12a2^βKO^* and control mice (means ± SE; *n* > 10; *P < 0.01). *C*: representative hematoxylin-eosin-stained retroperitoneal fat tissue images (×20) of 10- and 30-wk-old female *Slc12a2^βKO^* and control mice. Scale bars = 50 µm. *D*: mean adipocyte cross-sectional area (μm^2^) morphometrically determined by analyzing retroperitoneal white fat tissue sections from 10- and 30-wk-old *Slc12a2^βKO^* and control mice (*n* = 3). Each point represents the mean adipocyte area found in single nonoverlapping digital image tissue sections taken at ×200 magnification (*n* > 27; *P < 0.001). *E* and *F*: plasma glycerol (*E*; mg/L) and triglycerides (*F*; TG mg/dl) of 16-h fasted 10- and 30-wk-old female *Slc12a2^βKO^* and control mice (means ± SE; *n* = 3; *P < 0.01).

The results shown in Fig. 1*A* demonstrate that 30-wk-old female mice are significantly overweight, a phenotype shared with males of the same age (41). However, unlike males, which develop overweight at ∼15 wk of age (41), BW gained by female *Slc12a2^βKO^* mice at 20 wk of age was indistinguishable from that of control mice (Supplemental Fig. S1*A*). Fasting plasma insulin (Fig. 1*B*), blood glucose (Fig. 1*C*), glucose tolerance (Fig. 1, *D* and *E*), and insulin sensitivity (Fig. 1*F*) were all normal in 10-wk-old *Slc12a2^βKO^* mice. However, 30-wk-old *Slc12a2^βKO^* mice fed a chow diet showed increased fed and 6 h-fasted plasma insulin (Fig. 1*B*) in the context of normal blood glucose (Fig. 1*C*), glucose intolerance, and insulin resistance (Fig. 1, *D*–*F*). In addition, 30-wk-old female *Slc12a2^βKO^* mice showed a significant increase in total fat mass (Fig. 2, *A* and *B*) and adipocyte hypertrophy (Fig. 2, *C* and *D*). However, contrary to males (41), no histological evidence of steatosis/steatohepatitis could be found in female *Slc12a2^βKO^* mice younger than 33 wk (Supplemental Fig. S2).

Of note, 20-wk-old female *Slc12a2^βKO^* mice showed increased fed plasma insulin, whereas blood glucose, either fed or fasted, remained normal (Supplemental Fig. S1, *B* and *C*) and within the context of conserved glucose tolerance and reduced whole body insulin sensitivity (Supplemental Fig. S1, *D* and *E*). Therefore, insulin resistance is detected before the onset of overweight in female *Slc12a2^βKO^* mice, unlike males (41). However, like males, 10-wk-old female *Slc12a2^βKO^* mice have increased plasma glycerol and triglycerides (Fig. 2, *E* and *F*), indicating that altered lipid metabolism precedes the development of overweight and metabolic disturbances in both male and female *Slc12a2^βKO^* mice. Therefore, the fuel control of female *Slc12a2^βKO^* mice worsens in an age-dependent manner before the onset of overweight or paralleling it, a phenotype similar yet milder than that demonstrated in male *Slc12a2^βKO^* mice.

### Abnormal Feeding Pattern in *Slc12a2^βKO^* Mice

**Figure 3.**
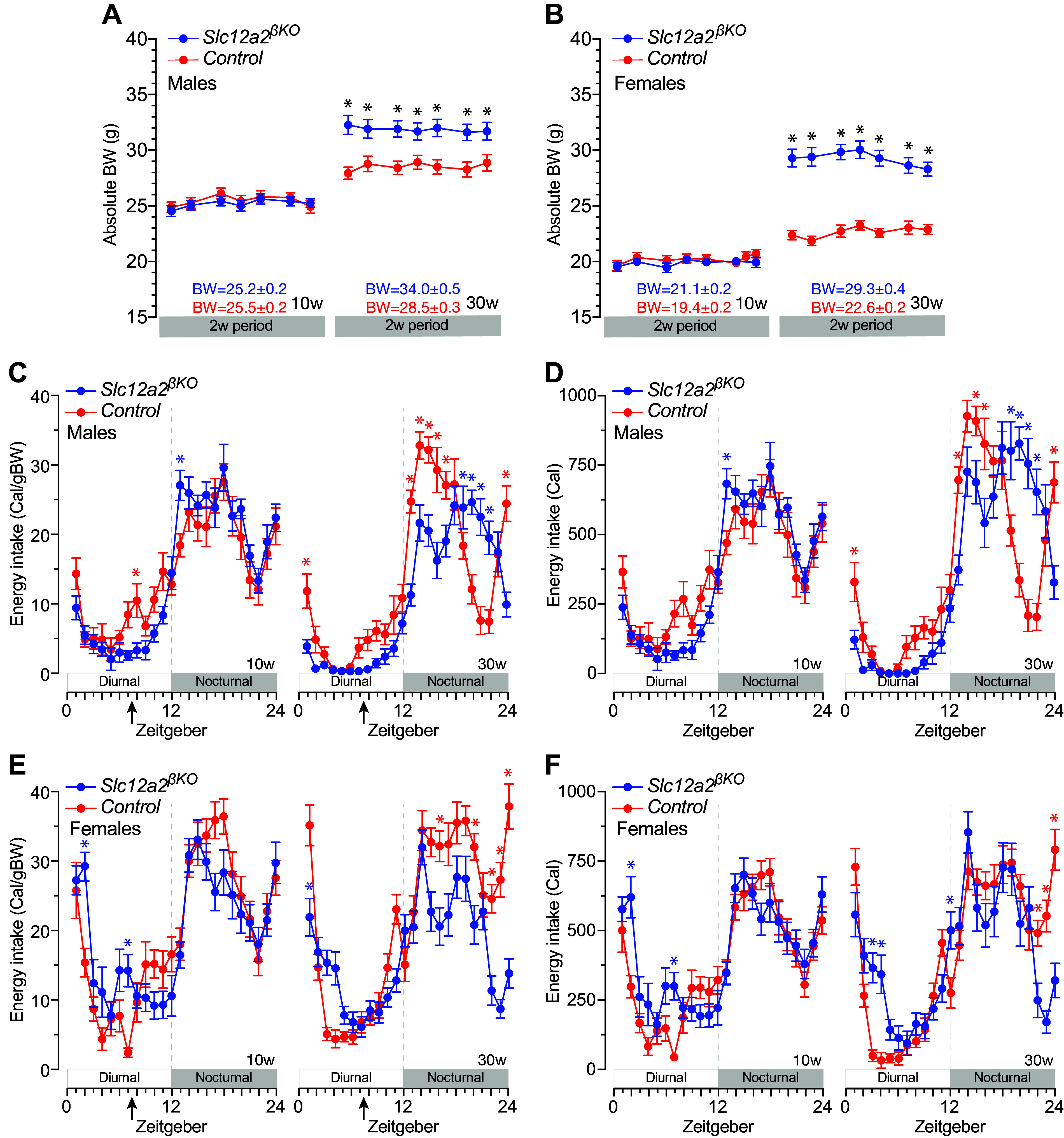
Circadian pattern of energy intake of *Slc12a2^βKO^* mice. *A* and *B*: body weight (BW) mass of 10-wk-old (10w) and 30-wk-old (30w) male (*A*) and female (*B*) mice fed ad libitum for 14 days. Each point represents the means ± SE (*n* = 9–10; *P < 0.05). *A* and *B*, *bottom*: mean BW values corresponding to male and female mice of the indicated ages and genotypes used to adjust net energy intake to BW. *C*–*F*: circadian pattern of energy intake normalized to BW (*C* and *E*; Cal/gBW) or net energy intake (*D* and *F*; Cal) of male (*C* and *D*) and female (*E* and *F*) *Slc12a2^βKO^* and control mice of the indicated ages. Each point indicates the means ± SE (*n* = 9–10; *P < 0.05). Zeitgeber 0 = 0630 h. Vertical arrows in *C* and *D* represent the time of the day when fed blood glucose was assayed.

**Figure 4.**
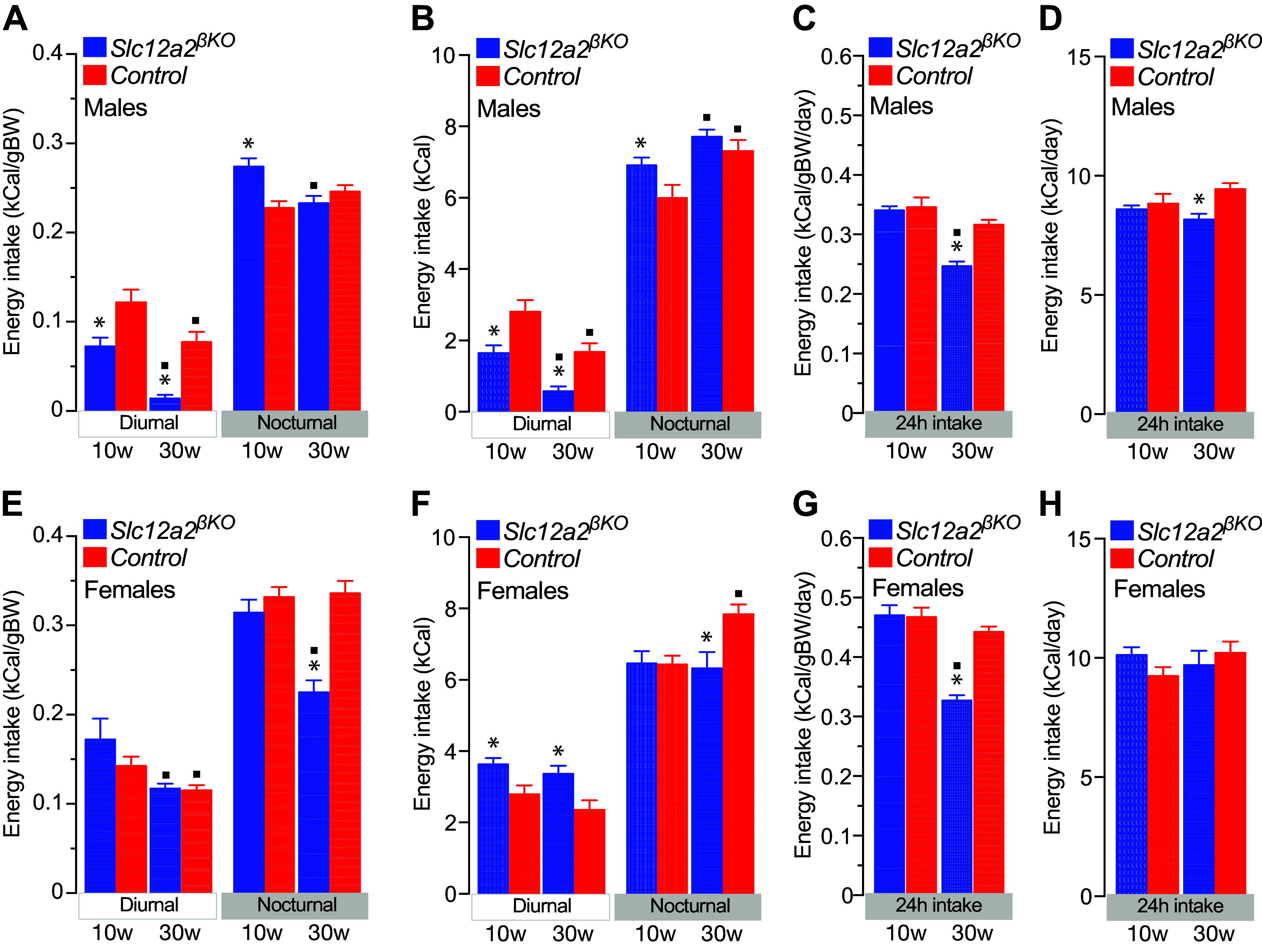
Adjusted and absolute energy intake of *Slc12a2^βKO^* mice. Mean adjusted or absolute nocturnal and diurnal integrated energy intake data of 10-wk-old (10w) and 30-wk (30w)-old male (*A*–*D*) and female (*E*–*H*) *Slc12a2^βKO^* and control mice. Feeding data are expressed adjusted to mean body weight (BW; *A* and *E*, kCal/gBW; or *C* and *G*, kCal/gBW/day) and in absolute terms (*B* and *F*, kCal; or *D* and *H*, kCal/day). Plotted are the means ± SE (*n* = 9–10; *P < 0.05 genotype; ^▪^*P* < 0.05 age).

To determine a potential relationship between BW gain and food intake, we next studied the feeding and behavioral patterns of energy intake in undisturbed *Slc12a2^βKO^* mice for 14 consecutive days. Importantly, the mean BW mass of 10- and 30-wk-old *Slc12a2^βKO^* mice of both sexes was maintained during the 2-wk study period of ad libitum feeding (Fig. 3, *A* and *B*). Therefore, calorie intake data were adjusted to BW or expressed in absolute values to avoid the general assumption that food intake is proportional to BW (48). The results shown in Fig. 3, *C*–*F*, and Supplemental Fig. 3, *A*–*D*, demonstrate minimal shifts in the circadian pattern of food intake in 10- to 30-wk-old male and female *Slc12a2^βKO^* mice. However, the integrated diurnal/nocturnal feeding data indicate that male *Slc12a2^βKO^* mice eat less food during the diurnal photoperiod of the day (Fig. 4, *A* and *B*, and Supplemental Fig. 3, *E* and *F*), contrasting the data of female *Slc12a2^βKO^* mice that consumed comparable amounts of food adjusted to BW at 10 to 20 wk of age (Fig. 4*E* and Supplemental Fig. 3*E*) or more in absolute terms at all ages studied (Fig. 4*F* and Supplemental Fig. 3*F*). Notably, 30-wk-old female *Slc12a2^βKO^* ate significantly less food than age-matched control mice only during the nocturnal active phase of the day, irrespective of BW adjustment (Fig. 4, *E* and *F*). Overall, and on a daily basis, the integrated feeding pattern of *Slc12a2^βKO^* mice did not result in an age-dependent increase in their normalized (Fig. 4, *C* and *G*) or absolute energy intake (Fig. 4, *D* and *H*, and Supplemental Fig. S3, *G* and *H*). Importantly, lean 10-wk-old male *Slc12a2^βKO^* mice ate significantly more food during the night (Fig. 4, *A* and *B*) without impacting daily energy intake in absolute terms or adjusted to BW (Fig. 4, *C* and *D*). However, overweight 30-wk-old male (Fig. 4, *C* and *D*) and female (Fig. 4, *G* and *H*) *Slc12a2^βKO^* mice ate similar or less per day than control. Therefore, lean 10-wk-old *Slc12a2^βKO^* mice consume normal net daily calories or per unit of BW, whereas overweight 30-wk-old *Slc12a2^βKO^* mice accumulate more BW per unit of calorie consumed than control mice. These data imply that the age-dependent overweight of *Slc12a2^βKO^* mice cannot be attributed to increased adjusted, net, or cumulative (Supplemental Fig. S4) energy intake.

### Age-Dependent Worsening of Meal Termination Control and Hastened Feeding in *Slc12a2^βKO^* Mice

**Figure 5.**
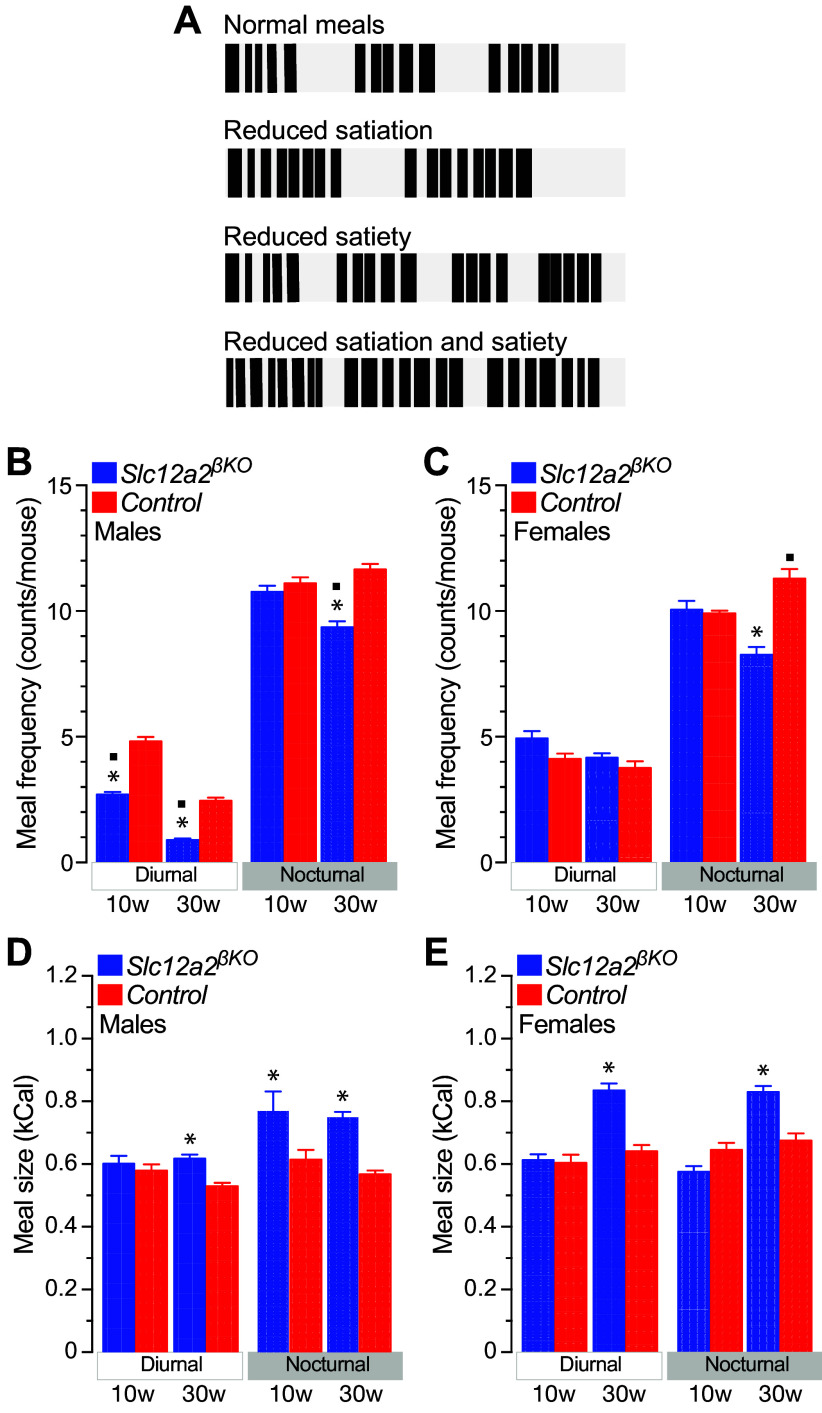
Satiation responses of *Slc12a2^βKO^* mice fed ad libitum a chow diet. *A*: behavioral representation of the dynamics of satiation (meal size changes) and satiety (intermeal interval changes) in responses to ad libitum eating. Each meal of a given size is a cluster of feeding bouts (filled boxes) separated by the next meal by a nonfeeding intermeal interval. By definition, satiation terminates meals, reducing their size, whereas satiet*y* prolongs the time spent between meals, usually, but not necessarily, reducing meal frequency. *B* and *C*: shown are the absolute number of meals eaten by male (*B*) and female (*C*) *Slc12a2^βKO^* and control mice of the indicated ages. Results are expressed as means ± SE (counts per mouse; *n* = 9–10; *P < 0.05). *D* and *E*: mean meal size (kCal) of 10-wk-old (10w) and 30-wk-old (30w) male (*D*) and female (*E*) mice. Data represent the means ± SE (*n* = 9–10; *P < 0.05 genotype; ^▪^*P* < 0.05 age). Data were continuously recorded for 14 days and distributed according to the diurnal and nocturnal photoperiods of the day.

To gain further insights into potential behavioral feeding mechanisms involved in *Slc12a2^βKO^* BW mass gain, we measured the satiation and satiety responses to ad libitum food intake (Fig. 5*A*) by following a validated paradigm (47). The data shown in Fig. 5*B* demonstrate that male *Slc12a2^βKO^* mice eat fewer meals per day than controls (10 wk: 13.5 ± 0.3 vs. 15.9 ± 0.3 meals/mouse, *n* = 10, *P* < 0.01; 30 wk: 10.3 ± 0.2 vs. 15.7 ± 0.3 meals/mouse, *n* = 10, *P* < 0.001), most notably during the inactive phase of the day (10-wk-old males), a consequence of a significant drop in the number of meals eaten during both phases of the day (30-wk-old males, Fig. 5*B*). In the case of females, 10- and 30-wk-old *Slc12a2^βKO^* mice consume normal or less meals per day, respectively, than control mice (10 wk: 15.2 ± 0.4 vs. 14.1 ± 0.4 meals/mouse, *n* = 9; and 30 wk: 12.4 ± 0.2 vs. 15.0 ± 0.5 meals/mouse, *n* = 10, *P* < 0.01; Fig. 5*C*). Therefore, these data suggest that the reduced adjusted, or normal net daily energy intake of overweight 30-wk-old male (Fig. 4, *C* and *D*) and female (Fig. 4, *G* and *H*) *Slc12a2^βKO^* mice may be attributed, at least partly, to fewer meals eaten. Nevertheless, the reduced number of daily meals eaten by lean 10-wk-old male *Slc12a2^βKO^* mice cannot account for their normal daily energy intake either adjusted to BW (Fig. 4, *C* and *G*) or not (Fig. 4, *D* and *H*).

To account for that, we next measured the mean caloric size of each meal eaten by *Slc12a2^βKO^* mice. As expected, the data in Fig. 5, *D* and *E*, demonstrate that 10-wk-old male *Slc12a2^βKO^* mice, but not females of the same age, eat nocturnal meals of significantly larger size than controls. Consequently, by definition, 10-wk-old male *Slc12a2^βKO^* mice have reduced satiation responses to nocturnal feeding. In the case of 30-wk-old mice, both male and female *Slc12a2^βKO^* mice also showed reduced satiation during ad libitum feeding, because they consumed diurnal and nocturnal meals of significantly larger caloric content than controls (Fig. 5, *D* and *E*). Notably, overweight 30-wk-old female *Slc12a2^βKO^* mice ate significantly larger meals during the night than those eaten by lean 10-wk-old female mice of identical genotype. Therefore, together these data indicate that reduced satiation control of feeding precedes the development of overweight in male *Slc12a2^βKO^* mice, and it is worsened by it in both male and female 30-wk-old *Slc12a2^βKO^* mice.

Interestingly, 20-wk-old male and female *Slc12a2^βKO^* mice had comparable daily normalized or absolute energy intake (Supplemental Fig. S3, *G* and *H*) but at the expense of consuming significantly more meals per day (males: 16.2 ± 0.3 vs. 13.6 ± 0.3 meals/mouse, *n* = 9–10, *P* < 0.001; females: 14.2 ± 0.2 vs. 11.1 ± 0.3 meals/mouse, *n* = 9–11, *P* < 0.001; Supplemental Fig. S5*A*) of slightly reduced caloric content (males: 0.512 ± 0.010 vs. 0.529 ± 0.011 kCal, *n* = 9–10; females: 0.635 ± 0.032 vs. 0.685 ± 0.025 kCal, *n* = 9–11; Supplemental Fig. S5*B*). Note that the diurnal or nocturnal number of meals or their caloric content was not significantly different from controls. Therefore, these data unmask a potential compensatory feeding behavior against reduced satiation, i.e., 20-wk-old *Slc12a2^βKO^* mice increased their number of daily meals while normalizing their size without reducing daily energy intake (Supplemental Fig. S3, *G* and *H*).

### Compensatory Satiety Responses to Reduced Satiation in *Slc12a2^βKO^* Mice

**Figure 6.**
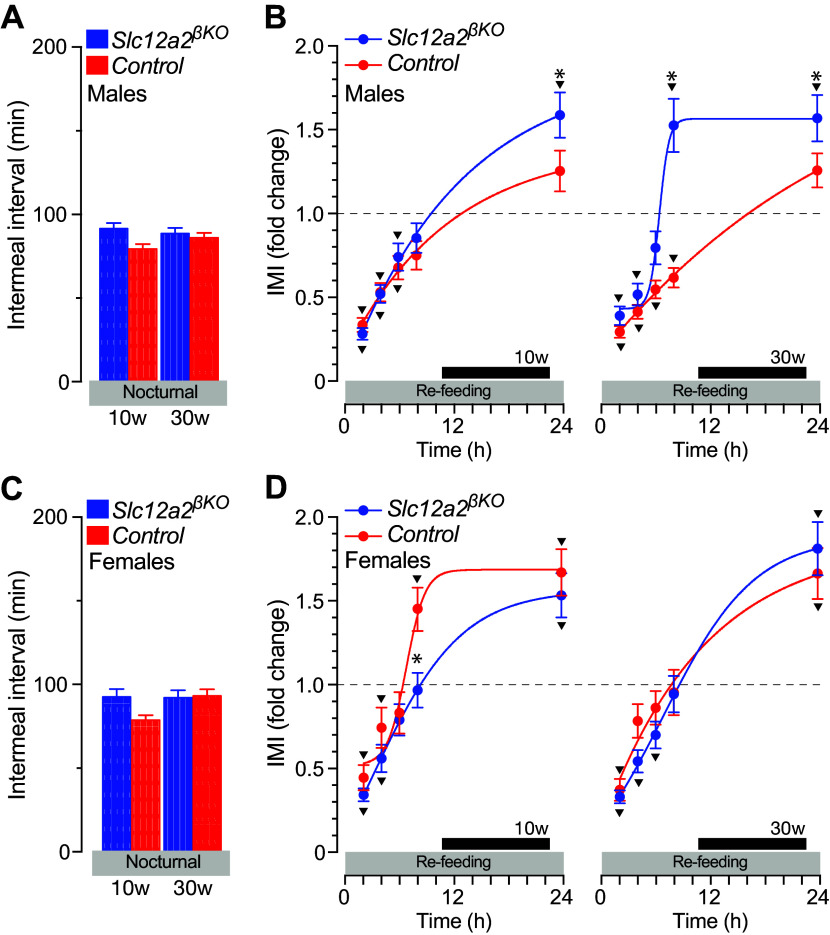
Satiety responses to ad libitum feeding and refeeding of *Slc12a2^βKO^* mice*. A* and *C*: mean nocturnal intermeal interval (IMI; min) was determined from the feeding microstructure of 10-wk-old (10w) and 30-wk-old (30w) male (*A*) and female (C) *Slc12a2^βKO^* and control mice. Data represents the means ± SE (*n* = 9–10, *P < 0.05) recorded during 14 consecutive nights (1830–0630 h). *B* and *D*: the dynamics of satiety responses to refeeding determined in 10- and 30-wk-old (30w) old male (*B*) and female (*D*) *Slc12a2^βKO^* and control mice allowed to refeed ad libitum after 16 h of fasting (1600–0800 h). The intermeal interval was continuously recorded every 2 h during 24 h to include the nocturnal photoperiod of the day (dark bar) when mice consume most of their food. Data represent the means ± SE relative to the baseline ad libitum condition represented by dashed lines (*n* = 9–10; *P < 0.05 genotype; ^▾^*P* < 0.05 baseline).

We have previously shown that the nocturnal ad libitum intermeal interval (IMI) of 10- to 30-wk-old C57BL/6J male mice is ∼70 min (47). In addition, the satiety (IMI) responses of mice to 16 h of fasting and during refeeding consist of at least three main components: *1*) a significant IMI shortening during the first 2 h of refeeding (>60% reduced satiety); *2*) a gradual feeding-dependent IMI recovery to reach ad libitum baseline levels within 6–8 h of refeeding; and *3*) a significant sex-dependent IMI lengthening above ad libitum baseline at the end of the scotophase indicating increased satiety (47). Consistently, the results in Fig. 6, *A* and *C*, demonstrate that 10- and 30-wk-old *Slc12a2^βKO^* and control mice spend comparable time between nocturnal meals while feeding ad libitum a standard chow diet (Supplemental Fig. S6*A*) demonstrating normal satiety responses. However, fasted male *Slc12a2^βKO^* mice showed increased (Fig. 6*B*) and both increased and hastened IMI kinetics at 10 and 30 wk of age (Fig. 6*B*, *right*), respectively, thus unmasking abnormal satiety responses to refeeding. Importantly, 10-wk-old female *Slc12a2^βKO^* mice showed faster satiety responses during refeeding, which were normalized in older mice (Fig. 6*D*; Supplemental Fig. S6*B*). Further, meal size during refeeding did not significantly differ from baseline ad libitum values in *Slc12a2^βKO^* mice (Supplemental Fig. S7*C*). Therefore, these results demonstrate that *1*) lean *Slc12a2^βKO^* mice have accentuated postprandial satiety responses to fasting, and *2*) the abnormal satiety responses of lean mice are maintained in overweight males, but not in females, independently of their impaired satiation responses to ad libitum feeding.

### Accelerated BW Mass Recovery in Fasted *Slc12a2^βKO^* Mice

**Figure 7.**
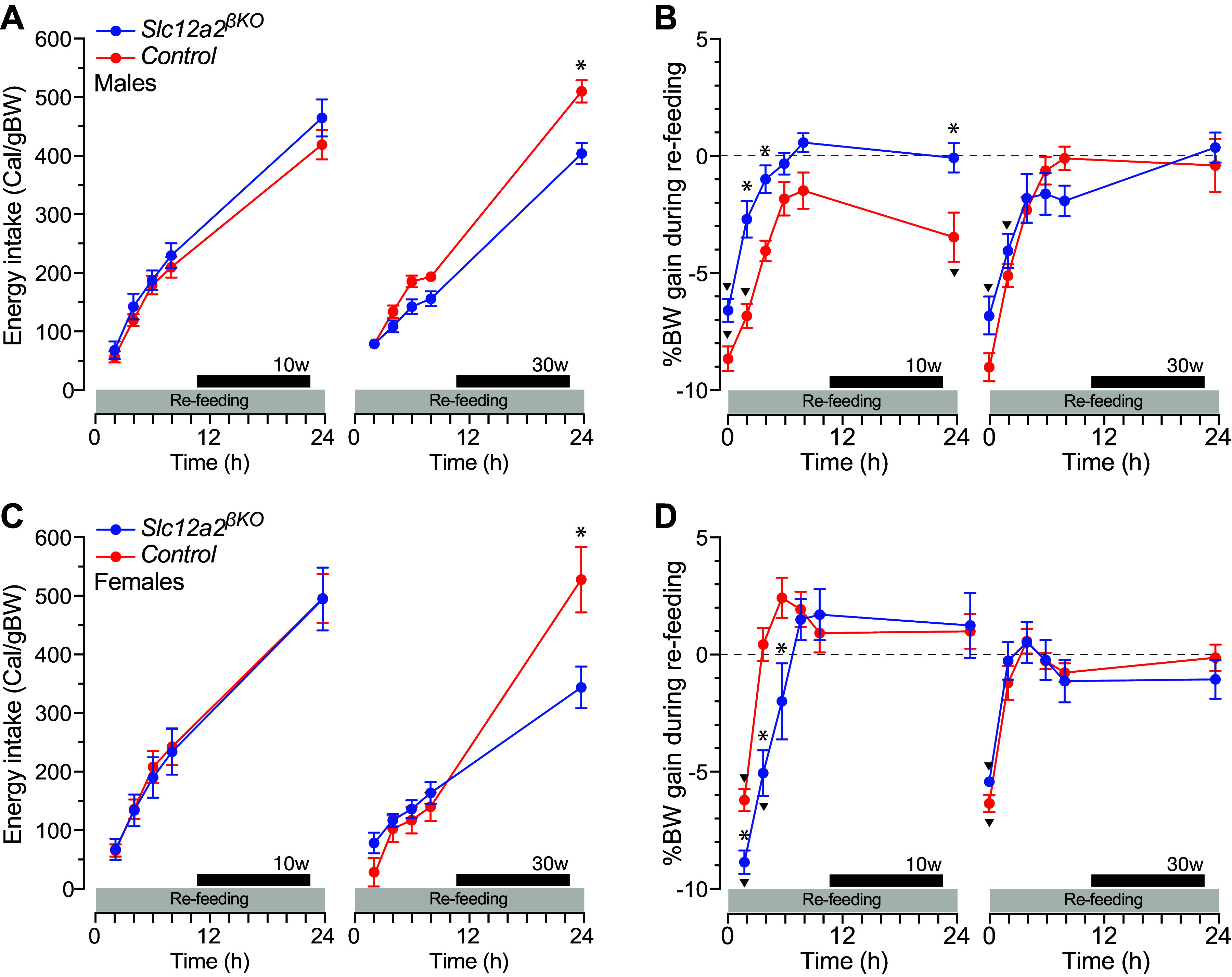
Energy intake and body weight (BW) gain during refeeding of *Slc12a2^βKO^* mice. *A* and *C*: normalized energy intake (Cal/gBW) of 10-wk-old (10w) and 30-wk-old (30w) male (*A*) and female (*C*) *Slc12a2^βKO^* and control mice recorded during 24 h of refeeding. Each point represents the means ± SE (*n* = 9–10; *P < 0.05 genotype; ^▾^*P* < 0.05 baseline ad libitum, represented by dashed lines for each genotype). *B* and *D*: body weight loss after 16 h of fasting and regained during refeeding of 10- and 30-wk-old male (*B*) and female (*D*) *Slc12a2^βKO^* and control mice. Data are shown as means ± SE (*n* = 9–10; *P < 0.05 genotype; ^▾^*P* < 0.05 baseline).

The results shown in Fig. 7, *A* and *C*, *left*, demonstrate that lean 16 h-fasted *Slc12a2^βKO^* and control mice consumed an equivalent number of calories per unit of BW during refeeding whereas 16-h-fasted overweight *Slc12a2^βKO^* mice ate significantly less than control (Fig. 7, *A* and *C*, *right*). In overweight mice, that reduction in postfasting energy intake was mostly accounted for by a lesser number of meals eaten and reduced rates at which *Slc12a2^βKO^* mice ate their meals relative to lean mice of identical genotype (Supplemental Fig. S7, *A* and *B*). Nevertheless, and despite eating less per unit of BW, overweight *Slc12a2^βKO^* mice fully recovered their lost mass at a pace identical to that of control mice (Fig. 7, *B* and *D*, *right*). However, lean male and female *Slc12a2^βKO^* mice regained their lost BW faster or slower, respectively, than controls (Fig. 7, *B* and *D*, *left*). Therefore, these data together support the hypothesis that the increased satiety responses to refeeding constitute a compensatory feeding mechanism triggered in response to reduced satiation.

### Hastened Feeding Behavior in *Slc12a2^βKO^* Mice

**Figure 8.**
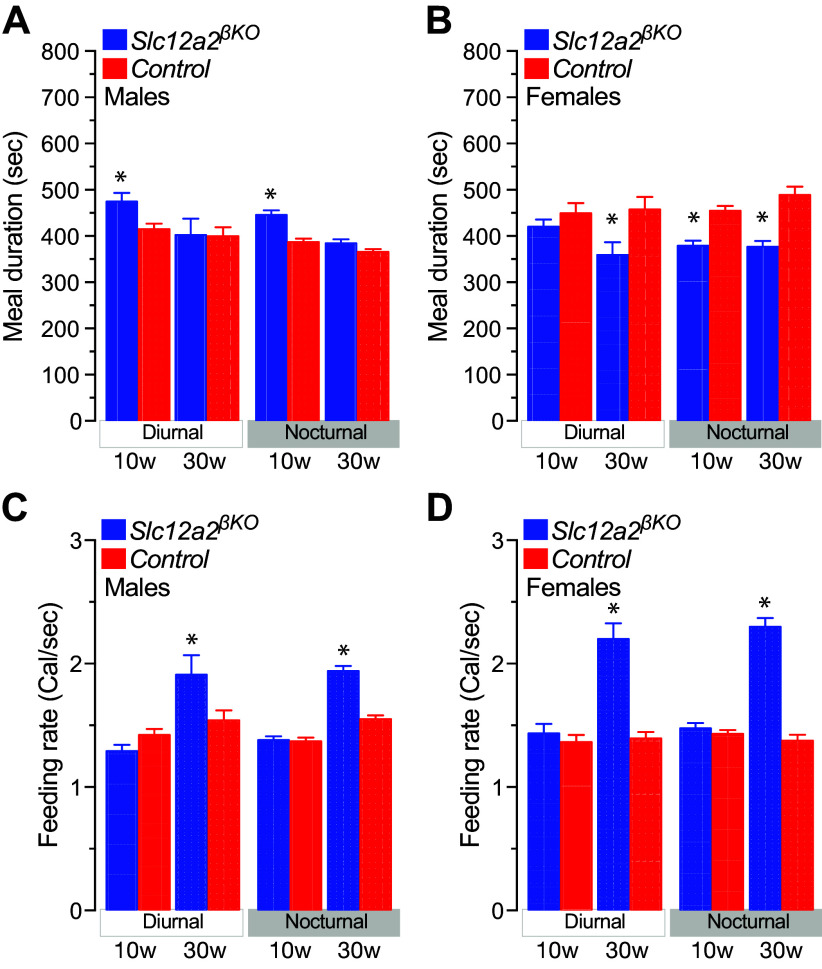
Time spent eating and rate of eating of *Slc12a2^βKO^* mice. *A* and *B*: time male (*A*) and female (*B*) *Slc12a2^βKO^* and control mice of the indicated ages spent (sec) eating their meals. Results are expressed as means ± SE (*n* = 9–10, *P < 0.05). *C* and *D*: rate (Cal/sec) at which 10-wk-old (10w) and 30-wk-old (30w) male (*C*) and female (*D*) mice ate their meals. Data represent the means ± SE (*n* = 9–10; *P < 0.05). Data were continuously recorded for 14 days and distributed according to the diurnal and nocturnal photoperiods of the day.

Fast eating is associated with overweight (49), and the latter has been proposed to promote fast eating (50–52). Therefore, we measured the time spent eating meals and the rate at which lean/overweight male and female *Slc12a2^βKO^* mice ate. The data shown in Fig. 8, *A*–*D*, demonstrate that lean 10-wk-old male and female *Slc12a2^βKO^* mice ate their nocturnal meals over a longer (Fig. 8*A*) or shorter (Fig. 8*B*) period of time, respectively, whilst both ate their meals at relatively normal rates (Fig. 8, *C* and *D*). Therefore, the time spent between individual bouts during a meal has sex-related differences. However, 30-wk-old *Slc12a2^βKO^* females, but not males, spent significantly less time eating their meals than control (Fig. 8, *A* and *B*). Therefore, overweight 30-wk-old male and female *Slc12a2^βKO^* mice consume meals of significantly larger caloric content (Fig. 5, *D* and *E*) and faster (Fig. 8, *C* and *D*) than control mice. Note that 20-wk-old male and female *Slc12a2^βKO^* mice spent less time eating their meals of normal caloric size resulting in increased feeding rates (Supplemental Fig. S5, *C* and *D*). Therefore, increased feeding rates can be considered as a response to overweight rather than its cause in this animal model.

In summary, the results presented here suggest that mice lacking *Slc12a2* in β-cells have impaired control of meal termination, which worsens paralleling BW gain and coexists with a gradual age-dependent compensatory increase in satiety. Since impaired satiation responses to feeding rather than increased absolute, adjusted to BW or cumulative daily energy intake antecede the onset of overweight and overweight mice eat comparable or less calories per day, we propose that altered satiation may play a potential causative role in the development of overweight and metabolic dysregulation whereas overweight may trigger compensatory satiety responses to attenuate/delay BW gain and metabolic dysregulation.

## DISCUSSION

We have recently demonstrated that male mice lacking *Slc12a2* in insulin-secreting β-cells develop overweight, hyperlipidemia, glucose intolerance, insulin resistance, and steatohepatitis spontaneously without overeating (41). These phenotypes, like those of other animal models of metabolic syndrome, were associated with impaired insulin secretory responses to exogenous glucose or fasting/refeeding cycles (53–55). We now extend these results by presenting evidence suggesting that female *Slc12a2^βKO^* mice also develop a similar metabolic phenotype to that of males, although milder (Figs. 1–2). However, contrary to males, insulin resistance in female *Slc12a2^βKO^* mice was detected at 20 wk of age before becoming overweight (Supplemental Fig. 1, *A* and *E*). Therefore, these data offered an opportunity to study a potential temporal relationship between feeding behavior, the development of insulin resistance, overweight, and metabolic abnormalities independently of excess net/adjusted calorie intake and BW gain. Nevertheless, it is important to consider that contrary to humans, obesity in rodents is difficult to define (56). In addition, the obesity-associated comorbidities found in male *Slc12a2^βKO^* mice were milder (41) than those typically observed in classic models of rodent obesity (57). Moreover, BW and the metabolic phenotype of 30-wk-old female *Slc12a2^βKO^* mice did not resemble that of typically obese female mice of similar ages (58).

It is generally accepted that overweight/obesity is caused by chronic hyperinsulinemia and an imbalance between energy intake and energy expenditure (59, 60). However, careful data analysis of published literature (61–67) also suggests that overweight or leanness may not necessarily follow parallel changes in daily net calorie intake or normalized to BW, even energy expenditure. In fact, BW gain without increased energy intake per unit of BW has been reported multiple times (68–72). Further, the *Fatzo/Pco* (MS-NASH) mouse model (53–55) and male *Slc12a2^βKO^* mice (41) developed a metabolic syndrome-like phenotype and nonalcoholic steatohepatitis in the absence of excess energy intake normalized to BW. Similarly, 24-h adjusted (Fig. 4, *C* and *G*) or net energy intake (Fig. 4, *D* and *H*), or cumulative food consumption (Supplemental Fig. S4) did not increase in overweight *Slc12a2^βKO^* mice, but it was rather reduced, particularly in males.

Consequently, what may drive BW mass accumulation and the metabolic phenotype of these animal models? Of the several potential alternatives involving the thermodynamics of energy balance, it has also been suggested that the meal pattern can modulate BW gain without net changes in daily caloric intake (73). Notably, obese *ob/ob* mice (74) and obese individuals (reviewed in Ref. 75) eat larger meals than lean controls. In addition, changes in meal size or frequency, rather than net daily caloric intake, are associated with overweight/obesity in rats (76) and in humans (77). A key concept stemming from our experiments relates to the age-dependent worsening of satiation (meal size) control observed in male *Slc12a2^βKO^* mice (Fig. 5, *D* and *E*). As a matter of fact, responses to terminate meals during ad libitum nocturnal feeding were significantly reduced in lean 10-wk-old male *Slc12a2^βKO^* relative to age-matched control mice (Fig. 5*D*). Since these mice had a mild reduction in islet secretory function (41), together these data support and extend recent results suggesting that nocturnal insulin responses to feeding reduce bout size in 10-wk-old C57BL/6J mice (34). Moreover, reduced satiation was present before differences in BW were significant and worsened in older male *Slc12a2^βKO^* mice, which showed further reduced secretory responses (41). Therefore, impaired islet secretion in response to feeding may precede the onset of overweight or obesity, whereas the behavioral control of feeding and secretory responses may be aggravated with BW gain. In other words, our data further the hypothesis that impaired insulin responses may reduce the satiation control of eating, implying that increased meal size may be a potential contributor to hastened BW gain and the overall metabolic phenotype demonstrated in male (41) and female *Slc12a2^βKO^* mice (Figs. 1–2 and Supplemental Fig. S1). However, meal size was normal in lean 10- to 20-wk-old female *Slc12a2^βKO^* mice (Fig. 5*E* and Supplemental Fig. 5*B*) but did increase at 30 wk of age suggesting that the satiation responses to feeding worsened in parallel to BW gain and after the development of whole body insulin resistance. Interestingly, some results have suggested that estrogens may reduce meal size and food intake (78–81) whereas others did not find such correlations (82). Nevertheless, we have shown that 10- to 30-wk-old female C57BL/6J mice eat more food than males and consume meals of higher caloric content than males during the night suggesting that female mice have higher age-independent thresholds for nocturnal satiation control than males (47).

At any rate, attributing overweight/obesity and the development of metabolic syndrome to increased meal size (reduced satiation) alone remains a difficult task. For instance, it is not known if the meal pattern per se affects energy expenditure, basal metabolic rate, and/or physical activity. Although interesting possibilities, all of them await experimental demonstration in any animal model of overweight/obesity. Intriguingly, 10-wk-old male and female *Slc12a2^βKO^* mice reduced their BW to a similar extent or significantly more, respectively, than control mice after 16 h of fasting (Fig. 7, *B* and *D*, *left*). However, control female mice lost less BW than control males, consistent with the fact that female mice, rats, and humans are more resistant to fat loss, tend to lose less BW, and may conserve more energy than males during calorie restriction, thus requiring significantly less energy to keep their BW (83–85). Therefore, our data suggest that the absence of *Slc12a2* in β-cells eliminated sex-related differences in energy expenditure, because BW loss in response to prolonged fasting is related to increased energy expenditure (86, 87). Moreover, *Slc12a2^βKO^* mice recovered their lost BW significantly faster (males) or slower (females) than control mice (Fig. 7, *B* and *D*, *left*) despite consuming an identical number of calories per unit of BW (Fig. 7, *A* and *C*, *left*). Therefore, together these data unmask potential sex-related differences in energy expenditure dynamics during/after fasting and refeeding in *Slc12a2^βKO^* mice.

When compared to mice models of spontaneous morbid obesity or diet-induced obesity, *Slc12a2^βKO^* mice developed overweight rather than obesity by consuming fewer calories of a chow diet. Since leptin reflects fat mass (88) and mouse models of diet-induced obesity usually retain central sensitivity to the long-term anorectic actions of leptin (89), it is possible that central leptin signaling in *Slc12a2^βKO^* mice remains intact as well. In line, we found that the reduction in 24-h energy intake observed in 30-wk-old overweight males (Fig. 4, *C* and *D*) and females (Fig. 4, *G* and *H*) *Slc12a2^βKO^* mice relates to ∼25% decline in the number of meals consumed per day (Fig. 5, *B* and *C*), despite eating the largest meals (Fig. 5, *D* and *E*). Interestingly, only male *Slc12a2^βKO^* mice showed a gradual age-dependent increase in satiety responses to refeeding without impacting the satiety control of ad libitum feeding (Fig. 6 and Supplemental Fig. 6). However, the role of leptin in the control of satiation and satiety remains difficult to conciliate. Indeed, high levels of leptin may have satiation effects (i.e., reduced meal size) as well (90, 91) or enhance satiation signals elicited by other hormones (92–94). However, recent work has suggested that physiological leptin may decrease energy intake by reducing meal frequency without significantly modulating meal size (34, 95). Contrary to earlier studies (90, 91), these data were obtained by using validated definitions of IMI/meal size, and therefore, leptin-associated meal pattern data can be interpreted according to the current behavioral definitions of satiation, i.e., processes that terminate a meal (reducing meal size), and of satiety, i.e., the noneating time spent between meals (modulating meal frequency independently of meal size) (96). Although leptin may have long-term satiety rather than satiation effects, the role of this hormone in the satiation and satiety control of energy intake requires further investigation.

While it is plausible that compensatory satiety responses are related to reduced satiation, at least in part, and/or to fat-derived leptin, it is important to recall that all islet hormones are directly or indirectly involved in the control of feeding (reviewed in Ref. 16). In the case of insulin, it has long been known that its plasma responses correlate with the caloric magnitude of meals in normal individuals (97). In addition, intraportal or intranasal insulin rapidly decreases meal size in rats (33) and in humans (98), respectively. Similarly, prandial insulin reduces bout size in mice (34) and meal size in rats (99). In addition, β-cell-derived amylin, which is cosecreted with insulin (100, 101) also reduces meal size, energy intake, and BW gain (102–104). Further, glucagon and glucagon-like peptide-1, as well as cholecystokinin and other hormones released in response to eating from islets and/or the gastrointestinal tract, and the actual state of fullness all participate in meal termination thus contributing to the overall satiation response to feeding (16, 105, 106).

In summary, together our results demonstrate that the metabolic dysfunction and abnormal feeding behavior of 10-wk-old male *Slc12a2^βKO^* mice represent an early phenotypic manifestation related to a primary defect in β-cells. In addition, since those phenotypes precede the onset of insulin resistance and BW gain, it follows that glucose intolerance, increased blood glucose, and plasma insulin, and the further worsening of the feeding control develop in parallel to BW gain in this animal model. In addition, our results may help explain, at least in part, the association that exists between *Slc12a2* gene polymorphisms and higher adiposity in humans (107–109), cattle (110, 111), and chickens (112).

## DATA AVAILABILITY

Datasets for Figs. 1–8 are available at https://doi.org/10.6084/m9.figshare.23589531.v1.

## SUPPLEMENTAL MATERIAL

10.6084/m9.figshare.23608698.v1 Supplemental Figs. S1–S7: https://doi.org/10.6084/m9.figshare.23608698.v1.

## GRANTS

This research has been supported in part by funds from the American Diabetes Association and the National Institutes of Health (1-17-IBS-258 and R21DK113446-01 to M.D.F.). The funders had no role in study design, data collection and analysis, decision to publish, or preparation of the manuscript.

## DISCLOSURES

No conflicts of interest, financial or otherwise, are declared by the authors.

## AUTHOR CONTRIBUTIONS

Y.D.R. and M.D.F. conceived and designed research; Y.D.R. and R.A. performed experiments; Y.D.R., R.A., and M.D.F. analyzed data; Y.D.R. and M.D.F. interpreted results of experiments; R.A. and M.D.F. prepared figures; M.D.F. drafted manuscript; Y.D.R., R.A., C.A.H., and M.D.F. edited and revised manuscript; Y.D.R., R.A., C.A.H., and M.D.F. approved final version of manuscript.
